# The COVID-related mental health load of neonatal healthcare professionals: a multicenter study in Italy

**DOI:** 10.1186/s13052-022-01305-7

**Published:** 2022-07-30

**Authors:** Luigi Gagliardi, Serena Grumi, Marzia Gentile, Roberta Cacciavellani, Giulia Placidi, Angelina Vaccaro, Claudia Maggi, Beatrice Gambi, Letizia Magi, Laura Crespin, Graziano Memmini, Marcello DeFilippo, Elena Verucci, Liliana Malandra, Laura Mele, Angelo Azzarà, Livio Provenzi

**Affiliations:** 1 Division of Neonatology and Pediatrics, Versilia Hospital, Azienda USL Toscana Nord Ovest, Viareggio, Pisa Italy; 2 Developmental Psychobiology Research Center , IRCCS Fondazione Mondino, Pavia, Italy; 3 Division of Neonatology, Azienza Ospedaliero-Universitaria di Pisa, Pisa, Italy; 4 Division of Neonatology and Pediatrics, Ospedale San Luca, AUSL Toscana NordOvest, Lucca, Italy; 5 Division of Neonatology and Pediatrics, Ospedale Lotti, AUSL Toscana NordOvest, Pontedera, Pisa Italy; 6 Division of Neonatology, Ospedale San Giovanni di Dio, AUSL Toscana Centro, Florence, Italy; 7 Division of Neonatology and Pediatrics, Ospedale San Donato, AUSL Toscana Sud Est, Arezzo, Italy; 8 Division of Neonatology and Pediatrics, Ospedale di Barga, AUSL Toscana Nord Ovest, Barga, Pisa Italy; 9 Division of Neonatology and Pediatrics, Nuovo Ospedale Apuano, AUSL Toscana NordOvest, Massa, Pisa Italy; 10 Division of Neonatology and Pediatrics, Ospedale di Grosseto, AUSL Toscana Sud Est, Grosseto, Italy; 11 Division of Neonatology and Pediatrics, Ospedale di Livorno, AUSL Toscana Nord Ovest, Livorno, Pisa Italy; 12 Division of Neonatology and Pediatrics, Ospedale di Cecina, AUSL Toscana Nord Ovest, Cecina, Pisa Italy; 13 Division of Neonatology and Pediatrics, Ospedale di Prato, AUSL Toscana Centro, Prato, Florence Italy; 14 Division of Neonatology, Azienda Ospedaliero-Universitaria Meyer, Florence, Italy; 15 Department of Brain and Behavioral Sciences, University of Pavia, Pavia, Italy

**Keywords:** Burnout, COVID-19, Healthcare professionals, NICU, Nurses, Stress

## Abstract

**Background:**

The COVID-19 pandemic has dramatically affected healthcare professionals’ lives. We investigated the potential mental health risk faced by healthcare professionals working in neonatal units in a multicentre cross-sectional observational study.

**Methods:**

We included all healthcare personnel of seven level-3 and six level-2 neonatal units in Tuscany, Italy. We measured the level of physical exposure to COVID-19 risk, self-reported pandemic-related stress, and mental health load outcomes (anxiety, depression, burnout, psychosomatic symptoms, and post-traumatic symptoms) using validated, self-administered, online questionnaires during the second pandemic wave in Italy (October 2020 to March 2021).

**Results:**

We analyzed 314 complete answers. Scores above the clinical cutoff were reported by 91% of participants for symptoms of anxiety, 29% for post-traumatic symptoms, 13% for burnout, and 3% for symptoms of depression. Moreover, 50% of the participants reported at least one psychosomatic symptom. Pandemic-related stress was significantly associated with all the measured mental health load outcomes, with an Odds Ratio of 3.31 (95% confidence interval: 1.87, 5.88) for clinically relevant anxiety, 2.46 (1.73, 3.49) for post-traumatic symptoms, 1.80 (1.17, 2.79) for emotional exhaustion, and 2.75 (1.05, 7.19) for depression. Female health care professionals displayed a greater risk of anxiety, and male health care professionals and nurses, of depressive symptoms.

**Conclusions:**

Despite the low direct clinical impact of COVID-19 in newborns, neonatal professionals, due to both living in a situation of uncertainty and personal exposure to contacts with parents and other relatives of the newborns, and having to carry out activities once routine and now fraught with uncertainty, displayed clear signs of mental health load outcomes. They must be considered a specific population at risk for psychological consequences during the pandemic.

## Background

Work-related stress is a real issue in the management of healthcare professionals. Those who are involved in complex, highly technological, and/or emotionally demanding contexts are known to be at higher risk for the development of mental health problems and burnout. Physicians, nurses and other healthcare professionals who work in neonatal and pediatric contexts are required to make timely and effective decisions which – in neonatal intensive care units (NICUs) – may often concern the safety and survival of very young at-risk patients [1, 2]. Moreover, in neonatal and pediatric contexts, the healthcare providers have the double mission to provide adequate quality of care to the infants and children as well as to their parents, which may further load on their cognitive and emotional resources and may lead – in the long run – to mental health issues and psychological problems [3, 4]. Previous studies have reported high levels of mental health problems in physicians, nurses and other healthcare specialists working in pediatric and neonatal settings. In the UK, a range between 37 and 61% of physicians and nurses reported high rates of burnout [5, 6] and similar rates have been reported in Europe (36%) [7] as well as in the United States (50%) and south America (41%) [8].

The symptoms reported by neonatal and pediatric healthcare professionals include a variety of mental health issues that may exacerbate into burnout and exhaustion. These include anxiety, depression, emotional exhaustion, psychosomatic symptoms, and post-traumatic stress [9–15]. Compared to their colleagues working in less critical environments (e.g., neonatal wards, NWs), physicians, nurses and other healthcare professionals who work in NICUs have been found to report more difficulties in managing the emotional and psychological issues related to their job. For example, in recent investigations up to 50% of NICU professionals have reported burnout symptoms due to workload and continuous experience of a physical stressful environment [16]. A recent study suggests that neonatal healthcare professionals may exhibit a profile of neuroendocrine dysregulation, with a flattened circadian rhythm of the hypothalamic-pituitary-adrenal axis [17].

During the 2020, the unprecedented COVID-19 pandemic has dramatically affected the mental health of citizens [18–21] and healthcare professionals [22–26]. Research to date has highlighted the relevance of Pandemic-related stress for the worsening of psychological symptoms in professionals who care for COVID-19 patients at the frontline of the healthcare emergency. Indeed, a systematic review and meta-analysis documented a high prevalence of stress, anxiety and depression beyond the clinical risk threshold [23]. Nonetheless, the potential traumatic consequences of the pandemic and of the related alteration of quality of life due to necessary mitigation strategies have been observed even in citizens and professionals who do not deal directly with severely ill COVID-19 patients. For example, a recent review including 62 studies from 17 countries reported a prevalence of 33 and 28% for anxiety and depression respectively, among general population and healthcare workers [24].

The effects of the COVID-19 pandemic for healthcare professionals working in NWs and NICUs have been poorly investigated to date. Though the clinical impact of COVID-19 in perinatology has been lower than in other medical specialties, healthcare professionals have seen their working routines profoundly overhauled, often in arbitrary ways [27]. As recently suggested [9], acute and chronic mental health problems in neonatal and pediatric healthcare professionals may lead to relevant impairment of the ability of the professionals to provide adequate quality of care for both the little patients and their families. The consequences may include diminished work effectiveness, decreased quality of care, poor communication with families and less efficient decision making [7]. From this point of view, the COVID-19 pandemic may be putting an additional toll on the psychological well-being of NWs and NICU professionals, resulting in a relevant mental health load with potential detrimental consequences. A recent report about neonatal healthcare workers’ well-being showed a significant worsening, with more than the 60% of the sample exhibiting emotional exhaustion and only a third of it reporting sufficient institutional strategies to meet these emotional challenges [28].

It is important for both professionals and healthcare systems to adequately measure and report on this mental health risk to promote appropriate preventive and therapeutic actions. As such, between October 2020 and March 2021(i.e., during the second pandemic wave in Italy) we launched the “Staff and Parental Adjustment to COVID-19 Epidemics – Neonatal Experience in Tuscany” (SPACE-NET) survey, with the aim of documenting the potential mental health risk faced by neonatal and pediatric healthcare professionals in Italy. In the present study we report on the mental health load experienced by physicians, nurses and other healthcare professionals who work in neonatal and pediatric NWs and NICUs.

## Methods

### Participants and procedures

The SPACE-NET project is a multicentre cross-sectional observational study that included 7 level-3 neonatal units (that is, units that provide care including intensive care to newborns < 32 weeks gestation or < 1500 g birth weight) in Tuscany, and all 6 level-2 neonatal units (that provide care to infants ≥32 weeks or > 1500 g, and no intensive care) of AUSL Toscana Nord Ovest. All the healthcare professionals working in NWs and NICUs were contacted by email. The sample was composed in 90.8% by women. Those who participated in the survey provided an informed consent and anonymously filled in a series of questionnaires aimed at assessing their emotional stress response to the COVID-19 healthcare emergency as well as a series of potential mental health outcomes including emotional exhaustion (burnout), depression, anxiety, psychosomatic and post-traumatic symptoms. The study has been approved by the Ethics Committee of the participant parties. All methods were carried out in accordance with the ethical standards as laid down in the 1964 Declaration of Helsinki and its later amendments.

### Measures

#### Socio-demographics

The socio-demographic and professional characteristics collected were sex, age (years), setting (NW or NICU), job (physician, nurse, or other, including psychologists, social workers, residents, midwives, physiotherapists, rehabilitation technicians, and auxiliary healthcare assistants), and job experience (years).

#### COVID-19 exposure and pandemic-related stress

The participants’ direct (own infection or risk of infection) or indirect (infection or risk of infection of significant others) physical exposure to SARS-CoV2 was assessed with an ad-hoc 5-item questionnaire previously used by our group [29]. The items of this questionnaire were rated dichotomously. A global index was obtained by summing all the responses and re-coding the sum into a *COVID-19 exposure* variable coded as “no” if sum was equal to 0 or “yes” if sum was above 0. The pandemic-related stress response was assessed using an ad-hoc 6-item questionnaire previously used by our group [30]. The items were rated 1 (low stress) to 5 (high stress). A mean global index was computed and used in this study as *pandemic-related stress index*.

#### Mental health outcomes

The symptoms related to the following domains of mental health status were investigated: depression, anxiety, psychosomatic symptoms, emotional exhaustion, and post-traumatic symptoms. Depressive symptoms were measured using the Beck Depression Inventory – II (BDI-II) [31], a 21-item scale widely used to assess subclinical and clinical depressive symptomatology. Each BDI-II item is rated on a 4-point Likert scale and a global sum score (*depressive symptoms*) is obtained with severe depression indexed by scores higher than 28. Anxious symptoms were rated using the state anxiety subscale of the State-Trait Anxiety Inventory – Y form (STAI-Y) [32]. The state anxiety subscale features 20 4-point Likert items that are summed up in global score (*anxious symptoms*); scores higher than 40 indicate a risk for clinically relevant anxiety. A list of psychosomatic symptoms obtained from the Psychosomatic Symptom Checklist [33] was rated by each participant for severity on a 6-point Likert scale (1 = low, 6 = high). A mean score was obtained to index *psychosomatic symptoms*. A subscale of the Maslach Burnout Inventory [34] measuring *emotional exhaustion* was also included. This subscale includes nine 7-point Likert scale items (1 = low, 7 = high). A global *emotional exhaustion* score is obtained by summing the items ratings and it indexes clinical symptoms if higher than 30. The Impact of Event Scale (IES) [35] was used to assess the *post-traumatic symptoms*. The IES is a 22-item questionnaire. Each item is rated on a 5-point Likert scale and a global sum score is obtained; scores higher than 33 are meant to suggest the presence of clinical risk.

### Statistical analysis

Univariate analyses were carried out using analysis of variance or independent-sample *t*-tests. The linear association between *pandemic-related stress index* and mental health outcomes was assessed by means of Pearson’s bivariate correlations. For variables for which a validated clinical severity cutoff is available (depression, anxiety, burnout, post-traumatic symptoms), a dichotomous variable [above cutoff/below cutoff] was computed, and binomial regressions were used to estimate the probability of scoring above cutoff, including the following predictors in the model: setting, job, job experience, gender and *pandemic-related stress index*.

As all mental health domains were correlated, to reduce the number of statistical comparisons and obtain an overall index, we used a principal component analysis to calculate a global *mental health load index* (*MHLI*) that would explain the largest portion of variance in mental health outcomes (i.e., *depressive symptoms*, *anxious symptoms*, *psychosomatic symptoms*, *post-traumatic symptoms*, and *emotional exhaustion*). For this analysis we set the minimum Eigenvalue to 1 and we adopted a non-rotated solution. We used the principal component with the highest loading and that explained the highest portion of variance as the primary outcome variable for the study. The MHLI has mean = 0 and standard deviation = 1.

Specific mental health outcomes were further tested as additional endpoints. The statistical analyses were conducted using R [36] and IBM SPSS Statistics for Windows, ver. 26.0 [37].

## Results

A total of 314 healthcare professionals participated in the study, out of 941 invited (32.9%). The majority were females (*n* = 281, 89.5%), reflecting the composition of the workforce of HCP invited to participate (90.8% in the source population), as is frequently observed in Italy in the neonatal wards. The sample studied was composed of physicians (*n* = 100, 31.8%), nurses (*n* = 145, 46.2%), and other professions (*n* = 68, of which 49 were midwives); 192 were working in NWs (61.1%) and 122 (38.5%) in NICUs. About half of the sample had a direct or indirect exposure to COVID-19, including a 10.5% who experienced the death of a friend or significant person. Healthcare professionals in NICUs reported higher *pandemic-related stress index* compared to NW counterparts, *(p* = .014), while no differences were seen between settings, jobs or sex Descriptive statistics are reported in Table 1.

**Table 1:** Descriptive statistics of the study sample

			Setting				Job					
	All (*n* = 314)		NWs (*n* = 192)		NICUs (*n* = 122)		Physicians (*n* = 100)		Nurses (*n* = 145)		Others (*n* = 69)	
	N	%	N	%	N	%	N	%	N	%	N	%
Sex												
Females	281	89.5	182	94.8	99	81.1	73	73.0	140	96.6	68	98.6
Males	33	10.5	10	5.2	22	18.0	27	27.0	5	3.4	1	1.4
COVID-19 exposure												
No	148	47.1	92	47.9	55	45.1	52	52.0	65	44.8	31	44.9
Yes	166	52.9	100	52.1	66	54.1	48	48.0	80	55.2	38	55.1
	Mean	SD	Mean	SD	Mean	SD	Mean	SD	Mean	SD	Mean	SD
Job experience (years)	18.74	10.61	19.34	10.55	17.78	10.73	17.13	10.57	22.37	9.46	13.42	10.27
Pandemic-related stress [1:5]	3.4	0.84	3.31	0.84	3.55	0.81	3.32	0.76	3.47	0.87	3.37	0.86

Note. [min:max]

The mental health status in the various domains investigated are shown in Table 2. Nurses reported higher *anxious symptoms* when compared to physicians, but not to other professionals, *p* = .041. Scores above the clinical cutoff were reported by 91% of participants for *anxious symptoms*, 29% of participants for *post-traumatic symptoms*, 13% of participants for *emotional exhaustion*, and 3% of participants for *depressive symptoms*. Moreover, 50% of the participants reported at least one psychosomatic symptom.

**Table 2:** Mental health outcomes for neonatal healthcare professionals

			Setting				Job					
	All		NWs		NICUs		Physicians		Nurses		Others	
	Mean	SD	Mean	SD	Mean	SD	Mean	SD	Mean	SD	Mean	SD
Symptoms of anxiety [20:80; 40]	54.68	10.55	54.05	9.97	55.82	11.31	52.5	10.73	55.85	10.72	55.41	9.52
Symptoms of depression [0:63; 28]	9.02	8.18	8.62	7.69	9.71	8.91	7.95	7.74	9.26	8.91	10.07	7.06
Psychosomatic symptoms [1:6]	2.04	0.95	1.95	0.90	2.18	1.00	1.92	0.88	2.09	1.02	2.10	0.87
Emotional exhaustion [1:54; 30]	16.46	11.25	16.8	11.51	15.97	10.88	17.77	11.52	15.5	10.85	16.57	11.64
Post-traumatic symptoms [0:88; 33]	25.75	15.75	24.61	15.07	27.64	16.69	23.74	14.98	27.13	16.22	25.77	15.73
Mental health load index (PC1)	0.00	1.00	−0.06	0.94	0.11	1.08	−0.12	0.97	0.05	1.06	0.07	0.91

Note. [min:max; clinical cutoff]; *PC1* principal component 1, *NWs* Neonatal Wards, *NICUs* Neonatal Intensive Care Units

We investigated the different response of females and males; the results show only slightly higher scores in females for depression (Table 3).

**Table 3:** Mental health outcomes in the studied sample according to gender

	Gender				
	Females		Males		P
	Mean	SD	Mean	SD	
Anxious symptoms [20:80; 40]	55.3	10.1	49.4	12.8	0.002
Depressive symptoms [0:63; 28]	9.0	7.7	9.2	11.9	0.87
Psychosomatic symptoms [1:6]	2.0	0.9	2.1	1.2	0.81
Emotional exhaustion [1:54; 30]	16.2	11.1	18.3	12.2	0.33
Post-traumatic symptoms [0:88; 33]	25.7	15.6	26.3	17.5	0.83
Mental health load index (PC1)	0.01	1	−0.05	1.3	0.75

In square brackets: [min:max; cutoff]. *PC1* principal component 1, *SD* standard deviation, *NWs* Neonatal wards, *NICU* Intensive care units

**Fig. 1:**
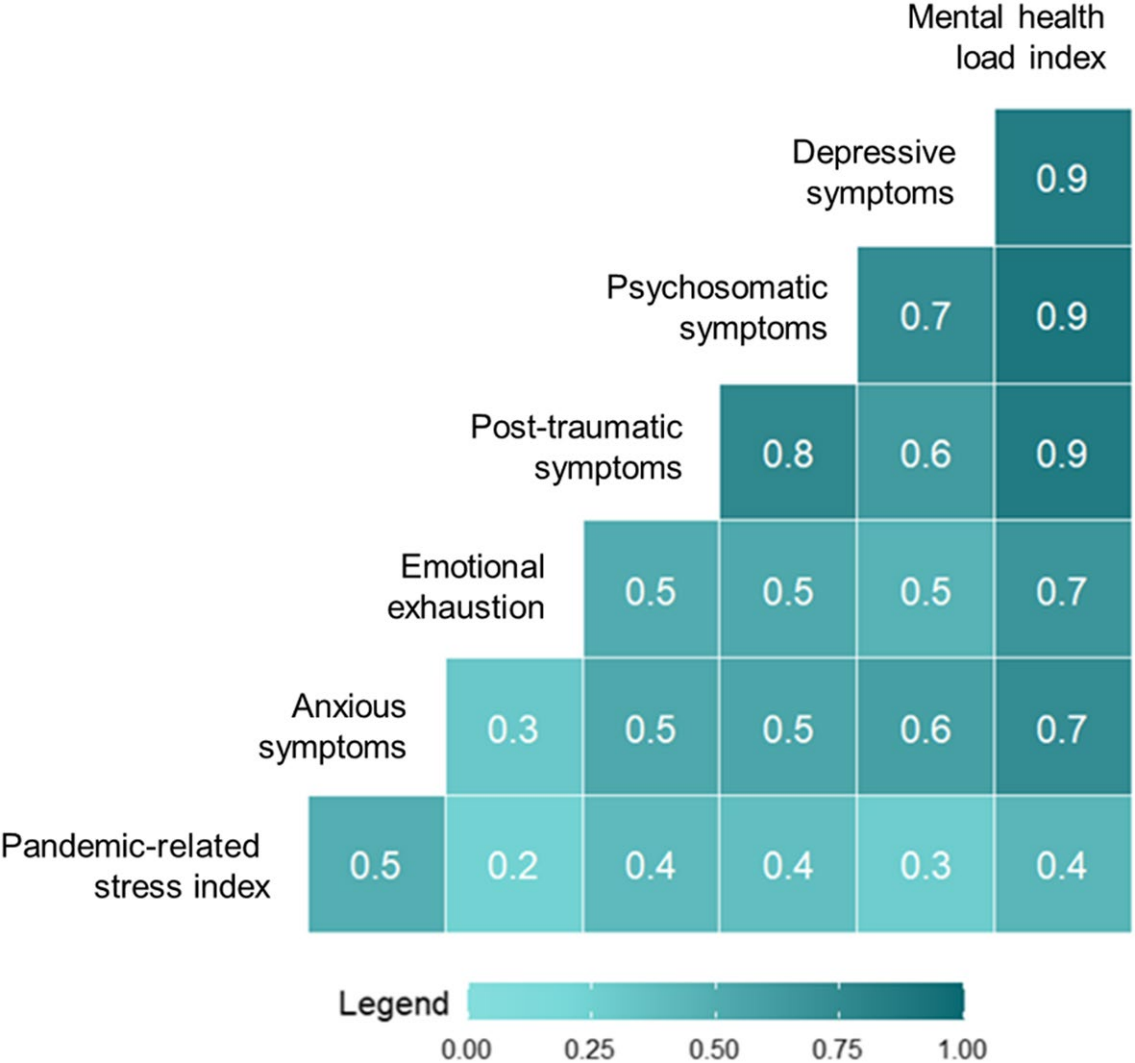
Bivariate correlations between pandemic-related stress and mental health outcomes

**Fig. 2:**
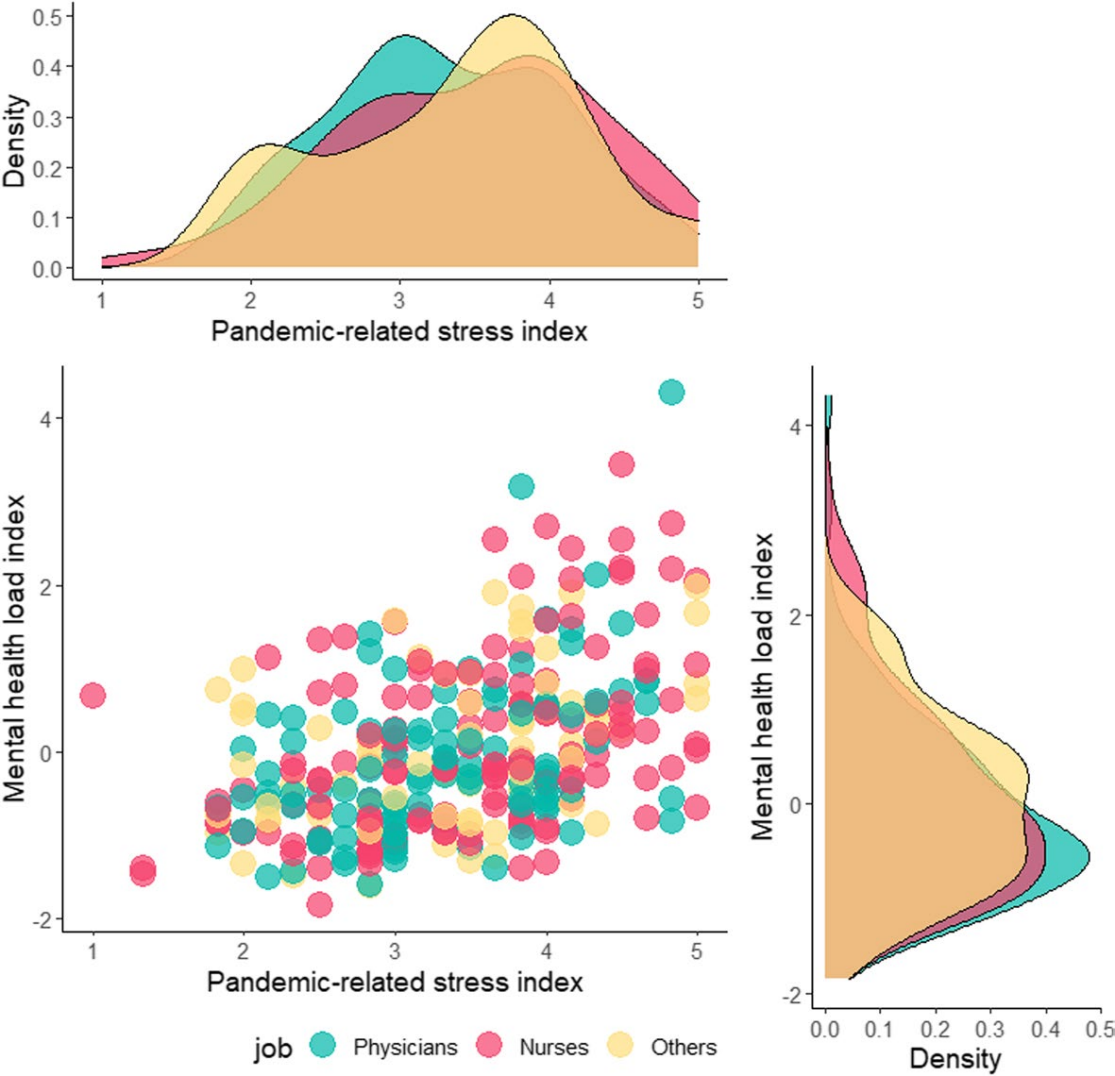
Association between pandemic-related stress and mental health load index split by setting

**Fig. 3:**
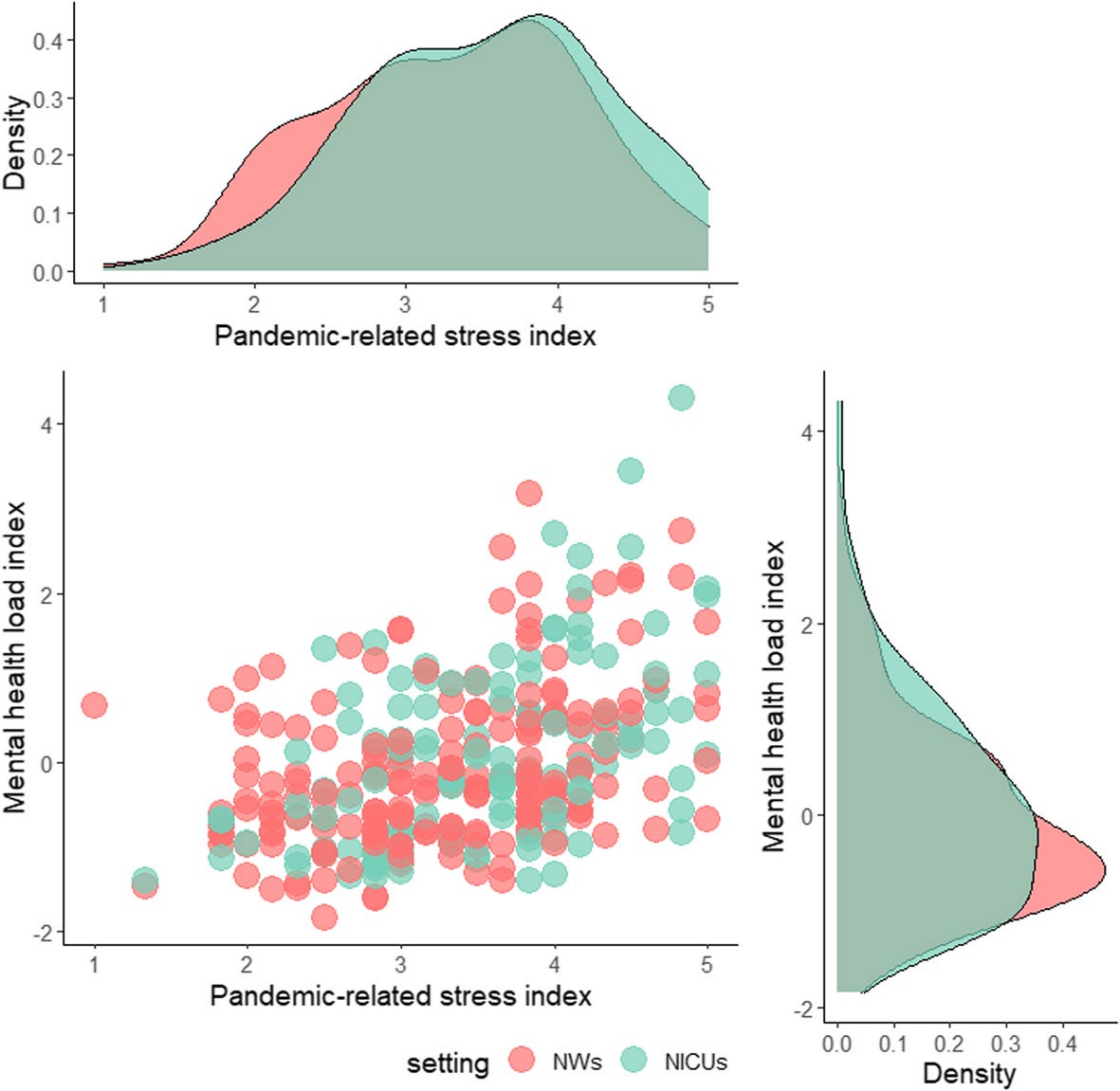
Association between pandemic-related stress and mental health load index split by job

The principal component analysis yielded a one-component solution, the *mental health load index*, explaining the 65.3% of total variance and with loadings ranging from 0.67 to 0.90. No statistically significant differences in *MHLI* emerged for job, setting and sex. *COVID-19 exposure* was not correlated with *MHLI* nor with any specific mental health load outcomes. *Pandemic-related stress index* significantly correlated with all mental health load outcomes as well as with the *MHLI* (Fig. 1). Figure 2 reports the association between *pandemic-related stress index* and *MHLI* by setting. Figure 3 reports the same association by job.

Logistic regressions, adjusting for setting, job and sex (Table 4) showed that an increase of one point in *pandemic-related stress index* was significantly associated with an increased risk of clinically relevant (i.e., above the accepted cutoff level) *anxious symptoms* (OR = 3.31; 95% CI 1.87, 5.88)*, post-traumatic symptoms* (OR = 2.46; 95% CI 1.73, 3.49),clinically relevant *emotional exhaustion (OR = 1.80, 95%CI* 1.17, 2.79*), and depressive symptoms* O*R* = 2.75, *95%CI* 1.05, 7.19). Interestingly, there were differences between men and women for the risk of both depression [higher risk in men: OR = 11.2, 95%CI 1.25, 100.8] and anxiety [higher risk in women: OR = 3.33, 95%CI 1.06,10.0], both in the complete sample, and when restricted to physicians only. No significant effects of setting, job, and job experience emerged for any of the outcomes except for a higher risk of depression in nurses.

**Table 4:** Association of COVID-related stress index with clinically significant outcomes. Logistic regression models

**A. Anxious symptoms**			
	*OR*	95% CI	*p*
COVID-related stress index	3.31	[1.87:5.88]	< .001
Setting (NWs)	Reference		
Setting (NICUs)	0.77	[.29,2.03]	0.60
Job (physician)	Reference		
Job (nurse)	1.42	[.51,3.94]	0.50
Job (other)	1.61	[.43,6.06]	0.43
Male sex	0.30	[0.10,0.94]	0.04
**B. Post-traumatic symptoms**			
	*OR*	95% CI	*p*
COVID-related stress index	2.46	[1.73,3.49]	< .001
Setting (NWs)	Reference		
Setting (NICUs)	1.44	[.80,2.60]	0.22
Job (physician)	Reference		
Job (nurse)	1.35	[.69,2.63]	0.38
Job (other)	1.41	[.63,3.14]	0.40
Male sex	1.80	[.72,1.55]	0.21
**C. Emotional exhaustion**			
	*OR*	95% CI	*p*
COVID-related stress index	1.80	[1.17,2.79]	0.008
Setting (NWs)	Reference		
Setting (NICUs)	0.53	[.24,1.20]	0.13
Job (physician)	Reference		
Job (nurse)	0.63	[.27,1.45]	0.28
Job (other)	0.75	[.24,1.20]	0.54
Male sex	1.44	[.45,4.56]	0.54
**D. Depressive symptoms**			
	*OR*	95% CI	*p*
COVID-related stress index	2.75	[1.05,7.19]	0.039
Setting (NWs)	Reference		
Setting (NICUs)	.66	[.15,2.88]	0.58
Job (physician)	Reference		
Job (nurse)	13.3	[1.09, 166.4]	0.045
Male sex	11.2	[1.25,100.8]	0.031

*RR* Risk Ratio, *NWs* neonatal wards, *NICU* neonatal intensive care units, *SE* standard error. Job (other) not included in the D model, as no healthcare professionals in this subgroup reported significant depressive symptoms above the clinical cutoff

For no investigated exposure (pandemic-related stress) or main outcomes (depression, anxiety, burnout, psychosomatic symptoms, post-traumatic symptoms, and MHLI) there was a relationship with date of response to the questionnaire.

## Discussion

The present study aimed at investigating the mental health load experienced by physicians, nurses and other healthcare professionals who work in NWs and NICUs during the COVID-19 pandemic. In our sample, more than 90% of participants reported anxious symptoms above the clinical cutoff, half of participants experienced at least one psychosomatic symptom and about one third of the sample reported a post-traumatic symptomatology above the clinical risk. These results suggest that also professionals in the perinatal field experienced the increased emotional burden documented for physicians working at the forefront of the pandemic during the COVID-19 emergency [18]. Moreover, the severity of pandemic-related stress largely impacted on their psychophysical health. Though very few professionals had had COVID-19, more than half of the sample experienced a direct exposure to the disease, including 10.5% who experienced the death of a significant other, and 27.4% a hospital admission. Thus, it is not unexpected that even if these professionals were not directly involved in the care of patients positive for COVID-19, these results were comparable to those reported for an Italian sample of frontline healthcare professionals [18]. Anxious symptoms especially emerged to be the more reactive outcome when facing critical situations, representing a sort of red flag of professionals’ mental health, perhaps also caused by the profound overhaul of established professional daily tasks.

Differently from the first published report on burnout experienced by NWs and NICUs workers [28], in our Italian context a limited percentage of NWs and NICUs professionals exhibited a level of emotional exhaustion compatible with a full-blown burnout. The effects of COVID-19-related stress on the depressive symptomatology were apparently limited, suggesting that depression may be a less reactive outcome during emergency crisis [38]. Nonetheless, it should be highlighted that we used the more restrictive BDI-II cutoff, which indicates the presence of severe depressive signs. It is also necessary to highlight the cross-sectional nature of the study that did not allow to disentangle the potential impact of pandemic-related stress from that of the usual workload. Therefore, it is not possible to exclude that the reported depressive symptoms may be a carry-over effect of difficulties related to the pre-pandemic period.

Physicians and nurses showed the same levels of physical COVID-19 exposure, independently of the type of unit (NW or NICU), while significant differences in pandemic-related stress and anxiety emerged for job and setting. In particular, professionals of NICUs exhibited a higher COVID-19-related stress that may be linked to the greater impact of containment measures on their professional practices. As for job, nurses exhibited higher levels of anxiety [39]. This difference emerged also in previous studies performed during the pre-pandemic period, showing a higher anxiety for nurses – especially if working in intensive units – linked to their work-related activities [39]. Therefore, this higher vulnerability may have been exacerbated during the lockdown period.

Our study has limitations. Firstly, the response rate of our survey (32.9%), though similar to that of another recent study [28], does not allow us to claim representativeness of our sample. The female-to-male ratio found in the sample, almost identical to that of the whole population of HCP working in these wards, is reassuring at least as far as sex selection is concerned. The high number of female HCP is expected in such categories as nurses, midwives, and doctors.

Secondly, the cross-sectional study design does not allow to assess the causal directions of the relationship between the pandemic-related stress and the professionals’ wellbeing. Moreover, the unavailability of pre-emergency data did not allow to disentangle the potential impact of pandemic-related stress from that of the usual workload. Although data collection occurred by self-report questionnaires, we used well-validated tools, except for the ad-hoc measure used to assess COVID-19 exposures and response. Finally, participants were enrolled from hospitals located in only one Italian region (i.e., Tuscany) that was not a primary hotspot of the virus spreading during the first lockdown. Nonetheless, Tuscany was dramatically hit during subsequent waves of the pandemic [40], as confirmed by the 10% of respondents who had experienced the death of at least one significant other and 30% of them who had indirect experience of a relative or close friend who needed intensive care hospitalization.

## Conclusions

The COVID-19 pandemic is likely to exert a relevant stress toll on the mental health of neonatal and pediatric healthcare professionals. Even if they are seldom directly involved in the care of patients positive for COVID-19, they should be considered as a specific population at risk for psychological consequences of the pandemic. As such, appropriate actions are needed from clinical institutions and policymakers to mobilize dedicated resources to take care of their psychological health.

## Data Availability

The raw data supporting the conclusions of this article will be made available by the authors, upon reasonable request.

## Abbreviations

NICU: Neonatal Intensive Care Unit
HCP: Health Care Professional
NW: Neonatal Ward
BDI: Beck Depression Inventory
STAI: State and Trait Anxiety Inventory
IES: Impact of Events Scale
MHLI: Mental Health Load Index
OR: Odds Ratio
CI: Confidence Interval
